# Recent Advances in Herbal Medicines for Digestive System Malignancies

**DOI:** 10.3389/fphar.2018.01249

**Published:** 2018-11-20

**Authors:** Jiyao Sheng, Xiaohan Zou, Ziqian Cheng, Yien Xiang, Wei Yang, Yang Lin, Ranji Cui

**Affiliations:** Jilin Provincial Key Laboratory on Molecular and Chemical Genetic, The Second Hospital of Jilin University, Changchun, China

**Keywords:** herbal medicine, digestive system malignancies, comprehensive treatment, chemotherapy, side effect

## Abstract

Herbal medicines, as an important part of traditional Chinese medicine (TCM), have been used to treat digestive system malignancies (DSM) for many years, and have gradually gained recognition worldwide. The role of herbal medicines in the comprehensive treatment of DSM is being improved from adjuvant treatment of the autologous immune function in cancer patients, to the treatment of both the symptoms and disease, direct inhibition of tumor cell growth and proliferation, and induction of tumor cell autophagy and apoptosis. Their specific mechanisms in these treatments are also being explored. The paper reviews the current anti-tumor mechanisms of TCM, including single herbal medicines, Chinese herbal formulations, Chinese medicine preparations and TCM extract, and their application in the comprehensive treatment of digestive system tumors, providing a reference for clinical application of TCM.

## Introduction

Digestive system malignancies (DSM) are a common cancer worldwide. They include esophageal, liver, pancreatic, gallbladder, gastric, and colorectal cancers. Due to their high incidence, rapid disease progression, and poor prognosis, they are a leading cause of death, and a public health burden around the world (Siegel et al., 2018). Of the available treatments for DSM, including surgery, radiotherapy, chemotherapy, targeted therapy, and immunotherapy, no current treatment can completely prevent tumor recurrence and metabolism (Tomasello et al., 2017; Finn et al., 2018; Iñarrairaegui et al., 2018; Toesca et al., 2018). Therefore, the development of new methods and drugs is particularly urgent for patients with DSM. Herbal medicine, as represented by traditional Chinese medicine (TCM), is an important component of complementary and alternative medicine, and has been developed in Asian countries, especially China.

Because of its potential for preventing and treating cancer, TCM has received wide attention from around the world, including Western countries. Many studies found that TCM combined with radiotherapy or chemotherapy for malignant tumors could significantly reduce the incidence of adverse reactions to radiotherapy or chemotherapy and improve cellular immunity and quality of life for patients (Qi et al., 2010; Ge et al., 2016; Liu et al., 2016a,b). Even some TCM preparations have a synergistic effect on chemotherapy, which can both prolong patient survival and reduce tumor recurrence and metastasis (Tian et al., 2012a; Pandey et al., 2015). Many studies have also shown that TCM could directly inhibit tumor cell growth and proliferation and directly or indirectly mediate their autophagy and apoptosis (Tang et al., 2009; Wang X. et al., 2012; Deng et al., 2013). Therefore, TCM, as an anti-tumor therapy, could play an important role in the treatment of DSM. In this paper, we mainly review the clinical application and possible mechanisms of herbal medicine in treating DSM, and consider their possible applications, to provide a reference for treatment of DSM.

## Survey methodology

PubMed was mainly used to search for related articles published using the keyword “herbal medicine,” “liver cancer,” “cholangiocarcinoma,” “hepatocellular carcinoma,” “gastrointestinal tumors,” “pancreatic cancer,” “gastric cancer,” “colon cancer,” “colorectal cancer,” “pancreatic cancer,” and “gallbladder cancer.” Then, screened articles were used as references for this review.

## Single herbal medicines for DSM

### Astragalus membranaceus

*Astragalus membranaceus* is a kind of tonifying TCM. It is often used in various advanced cancers to strengthen and consolidate body resistance (Li et al., 2017; Qi et al., 2017). Traditional Chinese medicine indications for *A. membranaceus* focus on “qi deficiency,” and patients often feel anorexia, a lack of strength, spontaneous sweating. Chemical extraction analysis shows that *A. membranaceus* contain many active components, such as astragalosides I–VIII, flavonoids, polysaccharides, amino acids and trace elements (Liu et al., 2013; Lu et al., 2016). Several clinical studies and animal experiments have confirmed that the effective components of *A. membranaceus* can enhance immune function and have anti-tumor effect (Cho and Leung, 2007; Huang et al., 2013; He et al., 2016; Li J. et al., 2016).

Astragalus polysaccharides are among the main active components of *A. membranaceus*, which are extracted with water from Astragalus roots (Xu et al., 2008). They have many biological functions such as immunomodulation, anti-inflammatory, antioxidants, anti-HBV, etc. (Dang et al., 2009; Huang et al., 2013; Yang et al., 2013; Zhang W. et al., 2017). Some studies have found that Astragalus polysaccharides can inhibit or kill tumor cells of digestive system (Tian et al., 2012b; Huang et al., 2016). Lai X et al. found that astragalus polysaccharides (100 mg·kg^−1^) could inhibit hepatoma tumor growth in H22 cell tumor-bearing mice. They also increase serum interleukin (IL) 2, IL-6, and tumor necrosis factor (TNF)-α concentrations (Lai et al., 2017), which suggests that astragalus polysaccharides play an anti-tumor role by regulating the immune system. The study also found that Astragalus polysaccharides could affect tumors by regulating the expression of Bcl-2 family proteins (up-regulated Bax, down-regulated Bcl-2; Lai et al., 2017). Notch receptors (Notch 1–4) are a kind of transmembrane protein receptor composed of extracellular domain, transmembrane domain and intracellular region. The main function of the extracellular domain is to bind specific ligands, and the intracellular region is mainly used for transcriptional regulation (Yoshida et al., 2013). Huang WH et al. found that, compared with normal tissues, mRNA expression and protein expression of Notch1 in HCC tissues increased significantly, and knockout of *Notch1* significantly inhibited the expression of apoptosis suppressor gene Bcl-2, and enhanced the expression of pro-apoptotic gene Bax, which indicates that astragalus polysaccharides may mediate apoptosis of human HCC cells through regulating Bcl-2 family proteins by inhibiting Notch1 (Huang et al., 2016). Some studies have found that astragalus polysaccharides can reduce adverse effects of chemotherapeutics while enhancing tumor cell sensitivity to them, thus functioning as an adjuvant therapy and improving therapeutic effects in DSM (Cao et al., 2014; Zhang D. et al., 2017).

Multidrug resistance (MDR) is a difficult problem in treating cancer; one cause of multidrug resistance is overexpression of P-glycoprotein (P- GP) in cells, which is encoded by the Multidrug Resistance 1 (*MDR1*) gene and acts as an efflux pump for various chemotherapeutics (Syed and Coumar, 2016). Tian et al. found that astragalus polysaccharides increased sensitivity of H22 HCC cell lines resistant to Adriamycin (H22/ADM cell lines) to chemotherapeutic agents, which may be related to downregulation of P-glycoprotein and *MDR1* mRNA expression (Tian et al., 2012a).

Astragalus saponins are active ingredients extracted from Astragalus roots, and mainly include astragalosides (I–VIII) and isomer iso-astragalosides (I, II) (Auyeung et al., 2010; Liu et al., 2010). Many recent studies suggest their potential value in the treatment of DSM (Li et al., 2017; Qi et al., 2017; Zhang S. et al., 2017). In a study of patients with advanced gastric cancer, Auyeung et al. found that astragalus saponins may mediate apoptosis in gastric cancer cells by activating caspase 3 and subsequently degradation of poly (ADP-ribose) polymerase. In addition, Astragalus saponins reportedly can induce stagnation of cell cycle of G_2_/M phase in gastric cancer cells and promote the down-regulation of the angiogenic protein vascular endothelial growth factor (VEGF) and metastatic proteins metalloproteinase (MMP), thus inhibiting tumor cell growth (Auyeung et al., 2012). This suggests that astragalus saponins may inhibit angiogenesis and metastasis of digestive system tumor cells. Wang et al. have found that astragalosides can combine with calpain inhibitor to induce endoplasmic reticulum stress-mediated apoptosis in colon cancer cells (Wang Y. et al., 2014). Wang T et al. found that astragalus saponins can inhibit the growth of human gastric cancer cell line BGC-823 cells *in vitro* and *vivo*, decrease its invasive ability (Wang T. et al., 2013). In addition, some studies have found that astragalosides can enhance the sensitivity of tumor cells to chemotherapeutic drugs (Auyeung et al., 2014; Xie et al., 2016; Ye et al., 2017). Wang et al. found Astragalus Saponin II could enhance the sensitivity of human HCC cells to 5- fluorouracil by suppressing autophagy via MAPK-mTOR pathway, thus improving the antitumor effects of chemotherapeutic drugs (Wang M. et al., 2017). These findings suggest that Astragalus saponins could be developed into an effective chemotherapeutic agent or adjuvant drug for the comprehensive treatment of DSM.

### Sophora flavescens

The dried root of *Sophora flavescens Ait* is used in TCM. Its active constituents include matrine, oxymatrine, sophocarpine, and oxysophocarpine (He et al., 2015; Ni et al., 2017). Basic studies found that alkaloids like matrine and oxymatrine could exert anti-tumor effects on various DSM, which has also been verified by many clinical studies and animal experiments (Zhou et al., 2014; Yong et al., 2015; Zhang and Yu, 2016; Wang X. et al., 2017).

Receptor-interacting protein-3(RIP3) is a serine–threonine kinase in the RIP kinase family, which can mediate the cell-death switch from apoptosis to necrosis in TNFα-induced necroptosis (Koo et al., 2015). Xu et al. found that matrine might promote expression of RIP3, thus promoting an anti-tumor effect in cholangiocarcinoma cells (Xu B. et al., 2017). The WNT signaling pathway is crucial for the generation and progression of both normal tissues and tumors (Pećina-Slaus, 2010). Ma Y et al. found that matrine (50 μg/ml) could inhibit migration and invasiveness of HPAC cells in pancreatic cancer. Moreover, expression of *MT1-MMP*, was reduced in matrine-treated HPAC cells, suggesting that matrine could be a pancreatic cancer treatment, through WNT-mediated down-regulation of MT1-MMP (Ma et al., 2015). Caspase-dependent apoptosis is a kind of programmed cell death. Wu et al. found that oxymatrine could mediate apoptosis in gallbladder cancer cells by activating caspase-3 and Bax and inhibiting Bcl-2 and NF-κB (Wu et al., 2014). Liang et al. found that oxymatrine regulated expression of epithelial-mesenchymal transition (EMT) markers such as E-cadherin, Snail and N-cadherin in colon cancer; expression of p65, a key protein in the NF-κB pathway was lowered at the same time (Liang and Huang, 2016). The results suggested that oxymatrine exerts an anti-tumor effect by down-regulating NF-κB, thus preventing EMT in colon cancer cells.

Moreover, studies found that matrine and oxymatrine could alleviate the toxicity of chemotherapeutic drugs, providing an adjuvant therapeutic approach for comprehensive treatment of digestive system malignancies (Liu et al., 2016b). The anti-tumor effect of gemcitabine on gallbladder carcinomas can be enhanced by suppressing the NF-κB pathway (Yang et al., 2012), which suggests that oxymatrine may increase efficacy of chemotherapeutic drugs by down-regulating the NF-κB pathway. Duan et al. found that the percentage of colon cancer cells suppressed with a combination of matrine and irinotecan (CPT-11), a first-line drug for colon cancer, was higher than that with either matrine or irinotecan alone, which might be associated with up-regulation of Topoisomerase (TOPO) I, Bax and Caspase-3 (Duan et al., 2017). The results suggested that matrine has a synergistic effect with irinotecan in the treatment of colon cancer. An experiment by Li et al. tested combined treatment with oxymatrine and 5-FU on human HCC both *in vitro* and *in vivo*, and found that tumor mass and volume in rats were more suppressed in the combined treatment group than in the single-drug groups (Liu et al., 2016a).

## Chinese herbal formulations for DSM

### Huangqin Tang/PHY906

Huangqin Tang, whose main components are *Scutellaria baicalensis, Paeonia lactiflora Pall, Glycyrrhiza uralensis Fisch*, and *Ziziphus jujuba Mill*, is a classical TCM preparation, first recorded in the book “Shang Han Lun” by Zhongjing Zhang during the Han Dynasty about 1800 years ago. It has been widely used to treating digestive system diseases that are accompanied with symptoms such as diarrhea, nausea, emesis and abdominal colic (Lam et al., 2010). PHY906, which is the improved version of Huangqin Tang, contains the same four herbs as Huangqin Tang but with a different weight ratio of 3:2:2:2 (Zhang et al., 2010). The application of PHY906 as an adjuvant for radiotherapy, chemotherapy, and targeted therapy in cancer has been explored by dozens of studies (Kummar et al., 2011; Liu and Cheng, 2012; Rockwell et al., 2013). Some studies confirmed the adjuvant role of PHY906 in treating DSMs, such as colon and pancreatic carcinoma (Farrell and Kummar, 2003; Yen et al., 2009; Kummar et al., 2011; Liu and Cheng, 2012; Saif et al., 2014). Lam et al. investigated PHY906 combined with irinotecan in a mouse colon cancer model (Lam et al., 2010). Overall survival in the PHY906 group was significantly better than in the control group; and intestinal mucosa in the control group was more severely damaged than in the PHY906 group, indicating that PHY906 decreases gastrointestinal damage caused by irinotecan. Yen Y et al. evaluated combined PHY906 and capecitabine for 42 patients with late-stage HCC who had lost the opportunity for resection (Yen et al., 2009). After two courses of treatment, 60% of patients showed stable or improved conditions; their median overall survival was 9.2 months with no significant quality of life reduction, implying that PHY906 could be used with capecitabine in treating HCC and providing support for a larger-cohort study. Saif et al. found that PHY906 significantly reduced nausea and emesis caused by capecitabine in 25 patients including 15 men and 10 women with pancreatic cancer in a Phase I clinical trial (Saif et al., 2014), which suggests that PHY906 combined with capecitabine is a safe substitute for gemcitabine for treating advanced pancreatic cancer.

Meanwhile, some studies investigated the mechanism of PHY906 in increasing the anti-tumor effect (Lam et al., 2015; Su et al., 2017). Sorafenib is the only drug approved by FDA for treating HCC, which inhibits the RAF/MEK/ERK pathways and tyrosine kinase receptors such as PDGF, VEGF and Kit (Wilhelm et al., 2004). Lam et al. discovered Huangqin and Shaoyao, which are the two main components of PHY906, might change the inflammatory state of tumor microenvironment and enhance the anti-tumor effect of sorefenib by inhibiting ERK1/2 phosphatase and thus increasing ERK1/2-P in HepG2 cells (Lam et al., 2015). Another study found that PHY906 could mediate apoptosis of colon cancer cells by regulating IFN-γ and activating responses toward steroid hormones, thus exerting an anti-tumor effect by protecting the epithelial barrier from being invaded by tumor cells (Su et al., 2017). The role of PHY906 in the comprehensive treatment of DSM warrants further research.

### Bu-Zhong-Yi-Qi-Tang

Bu-zhong-yi-qi-tang (also known as TJ-41) is a traditional medicine widely used in China, Japan and Korea. It contains 7 herbs, including *Scutellaria baicalensis, Pinellia tuber, Zizyphi fructus, Zingiberis rhizoma, Glycyrrhiza radix, Coptidis rhizoma*, and *Panax ginseng* (Qi et al., 2010). Reportedly, TJ-41 can decrease side effects and increase curative effects of radiotherapy and chemotherapy in treating DSM (Qi et al., 2010). Jeong et al. randomly divided 40 patients with liver-cancer into an experimental group who received two weeks' treatment with TJ-41, and a control group. They found that the experimental group had significantly less fatigue than did the control group (Jeong et al., 2010). Interleukin-6 (IL6) affects development of cachexia, which is a major cause of cancer-related death. Yae et al. found that macrophages and serum IL-6 expression were reduced, and cachexia alleviated, in mice with colon cancer treated by TJ-41(Yae et al., 2012). Kuo et al. found that TJ-41 enhanced toxicity of mitomycin C for human gastric adenocarcinoma MKN-74 cells, probably through a non-apoptotic mechanism (Kuo et al., 2014). Kao et al. found that TJ-41 mediated apoptosis by inducing stagnation of G_0_/G_1_ cell-cycle phases and inhibiting DNA synthesis, thus preventing proliferation of human Hep3B and HepG2 liver cancer cell (Kao et al., 2001).

### Shi-Quan-Da-Bu-Tang

Shi-quan-da-bu-tang (also known as TJ-48) is a TCM based on ten herbs: *Paeonia lactiflora, Poria cocos, A. membranaceus, Cinnamomum cassia, Glycyrrhiza inflata, Liqusticum wallichii, Angelica sinensis, Atractylodes macrocephala*, and *Rehmannia glutinosa* (Qi et al., 2010). Recently, some studies revealed TJ-48 might play a role in immune regulation and tumor treatment (Ikemoto et al., 2014). This medication can alleviate side effects caused by chemotherapy and radiotherapy during the treatment of DSM, and also prevent metastasis (Nishiuchi et al., 2013; Amitani et al., 2015). Ikemoto discovered TJ-48 could increase activity of T cells, based on decreased Foxp3^+^ Tregs in patients with advanced pancreatic cancer (Ikemoto et al., 2014). Another study indicated that TJ-48 might slow liver cancer progression and lengthen recurrence-free survival by suppressing Kupffer cell-mediated oxidative stress (Nishiuchi et al., 2013).

### Daikenchuto

Daikenchuto (TJ-100) is a traditional herbal medicine, also called Kampo, composed of *Capsicum Annuum*, dried ginger, ginseng and cerealose (Endo et al., 2017) and can be used for gastrointestinal diseases including intestinal obstruction and Crohn's disease (Kominato et al., 2016; Okada et al., 2016). Some studies found that TJ-100 can suppress tumor development and alleviate side effects of surgery (Yoshikawa et al., 2015; Nagata et al., 2016; Hasebe and Matsukawa, 2017). Hasebe et al. discovered TJ-100 can inhibit the downstream pathway activated by EGFR and effectively suppress tumor growth in a mouse model of colon cancer (Hasebe and Matsukawa, 2017). Yoshikawa et al. postoperatively treated 245 patients with gastric cancer who underwent total gastrectomy, with either TJ-100 or placebo (Yoshikawa et al., 2015). The result showed theTJ-100 group had a shorter median time to first bowel movement than the placebo group (94.7 vs. 113.9 h), and a lower incidence of gastrointestinal dysfunction at 12 days after surgery than did the placebo group.

## Traditional chinese medicine extract for DSM

### Cantharidin(C_10_H_12_O_4_) and norcantharidin(C_8_H_8_O_4_)

Mylabris has been used in cancer treatment for more than 2000 years (Lao et al., 2013; Zeng et al., 2016). Cantharidin (CTD) (Supplementary Figure 1), a terpenoid, is the main active ingredient of Mylabris, and it has a significantly cytotoxic effect on tumor cells (Kadioglu et al., 2014; Hsia et al., 2016). However, its clinical application is limited by strong irritation of the urinary system and digestive system when taken orally or intravenously (Wang G. et al., 2018). Norcantharidin (NTCD), a demethylated derivative of CTD, has relatively low toxicity and similarly antitumor activity to cantharidin (Puerto Galvis et al., 2013). Studies have shown that CTD and NCTD might mediate apoptosis to inhibit tumor development and have a metabolic impact on tumor cells (Rauh et al., 2007). CTD and NCTD were also shown to inhibit serine/threonine protein phosphatase 1 and serine/threonine protein phosphatase 2A (Bian et al., 2014). These terpenoids affect intracellular signal transduction and cell-cycle progression (Yeh et al., 2010).

Reportedly, CTD and NCTD inhibit DSM proliferation and metabolism, including liver cancer (Shen et al., 2015; Su et al., 2015). Le et al. found that CTD (5 μM) had an antitumor effect on HCC stem cells in a dose- and time-dependent manner, which may be associated with down-regulation of β-catenin and cyclin D1, and inhibition of cell self-renewal ability (Le et al., 2016). Shen et al. found that CTD inhibited invasiveness of pancreatic cancer cells by downregulating matrix metalloproteinase 2, which has a major role in remodeling extracellular matrix (Shen et al., 2015). Another study suggested that CTD might induce apoptosis of SGC-7901 and BGC-823 cells and G_2_/M phase arrest by activating Bcl-2 proteins in gastric cancer (Wang T. et al., 2015). Overexpression of family-with-sequence-similarity-46C (FAM46C) reportedly inhibits invasiveness of liver cancer cells by suppressing transforming growth factor-β-Smad signaling and EMT; *FAM46C*-knockout can change the anti-metastatic effect of NCTD on tumor cells, suggesting the anti-hepatoma impact of NCTD is affected by up-regulating FAM46C (Wan et al., 2017). MiR-214 is significantly down-regulated in HCC tissues, which is associated with low clinical progression and poor prognosis in HCC (Shih et al., 2012). Overexpression of miR-214 could inhibit growth and invasiveness in HCC (Wang J. et al., 2012, 2013). Lu S et al. found that NCTD could significantly inhibit tumor growth in liver cancer-bearing mice, and the inhibitory effect may be associated with enhanced anti-tumor activity of tumor-associated macrophages (Lu et al., 2014). In addition, NCTD significantly inhibited β-catenin expression, which could be reversed by miR-214 inhibitor (Lu et al., 2014).

Moreover, some studies have also found that combining radiotherapy or chemotherapy with CTD and NCTD could reduce the side effects of radiotherapy and chemoradiotherapy, and increase sensitivity of tumor cells to chemotherapeutic drugs, thus enhancing their efficacy (Sun et al., 2016; Zhang Y. et al., 2017). Wang et al. have found that CTD and NCTD might enhance the toxicity of gemcitabine and erlotinib for human pancreatic cancer cells by inhibiting the beta-catenin pathway, thus augmenting treatment of pancreatic cancer (Wang W. J. et al., 2015).

### Berberine(C_20_H_18_CLNO_4_)

Berberine (Supplementary Figure 2) is an isoquinoline alkaloid, extracted as a quaternary ammonium compound from *Coptis chinensis* (Huanglian in Chinese), *Hydrastis canadensis, Berberis aristata, Berberis vulgaris*, and *Berberis aquifolium* (Tang et al., 2009). Berberine is used for bringing down fevers and fighting intestinal bacterial infections due to its antibiotic effect. Berberine usually utilized in TCM to treat infectious diseases, including bacterial diarrhea, intestinal parasitic infection and ocular *Chlamydia trachomatis* infection, based on its robust resistance to bacteria, viruses, fungi, protozoa, worms and chlamydia (Tang et al., 2009). In the recent years, several studies have found that berberine might suppress tumor invasiveness and metastasis through multiple mechanisms (Wang et al., 2010; Liu et al., 2015). Yu et al. found that berberine could mediate apoptosis and autophagic death in HepG-2 cells through activation of AMPK, which is a kind of metabolic-sensing protein kinase (Yu et al., 2014). Wang et al. found that berberine (>100 μM) may inhibit HCC cell invasiveness and metastasis through up-regulation of plasminogen activator inhibitor-1 and down-regulation of urokinase-type plasminogen activator (Wang X. et al., 2016). Yi et al. found that berberine can significantly inhibit activation of the Akt pathway and inhibit the growth of tumors (Yi et al., 2015).

Other studies found that berberine could be combined with chemotherapeutics to neutralize their toxicity, thereby enhancing their anti-tumor effects (Pandey et al., 2015; You et al., 2016; Gong et al., 2017). Epidermal growth factor receptor (EGFR), a receptor for the ErbB family, is usually overexpressed in gastric cancer cells, which is associated with poor prognosis (Kim et al., 2008). Wang J et al. found that berberine can mediate apoptosis by inhibiting the EGFR pathway and enhance the efficacy of cetuximab or erlotinib in gastric cancer cell lines, which provide strong support for the potential of berberine in the treatment of DSM (Wang J. et al., 2016).

## Chinese medicine preparations for DSM

### Huaier granules

Huaier granules are a new anti-tumor drug extracted from *Trametes robiniophila Murr* (Huaier) (Zhao et al., 2017). Its main active ingredient is a proteoglycan composed of 41.53% polyose, 12.93% amino acid, and 8.72% H_2_O (Zou et al., 2015). The drug has been approved by (CFDA) the Chinese Food and Drug Administration for patients with malignancies, especially those with primary liver cancer who have lost the opportunity for surgery and chemotherapy (CFDA approval number, Z20000109; Bao et al., 2016). Various studies confirm that Huaier granules can inhibit tumor growth, promote tumor cell apoptosis, induce secretion of various cytokines, and increase immunity, while enhancing sensitivity to chemotherapeutics and reversing drug resistance. Because of the wide potential applications of Huaier granules, many studies have focused on its use and mechanisms for the treatment of DSM (Wang X. et al., 2012; Song et al., 2015).

Pathways for hypoxia-inducible factor (HIF)-1α, VEGF, RNA-binding factor 1(AUF-1), and astrocyte elevated gene-1 (AEG-1) play roles in the progression of liver cancer (Yang et al., 2014; Liu et al., 2017). Cong et al. did a series of *in-vivo* and *in-vitro* experiments with human HCC SMMC-7721 cells (Li C. et al., 2015). The results suggested that Huaier polysaccharide (TP-1) inhibits the above pathways by partially down-regulating HIF-1α, VEGF, AUF-1, and AEG-1 proteins, thereby suppressing revascularization, growth and metabolism of liver cancer cells. Mitogen-activated protein kinase (MAPK), which mediates cell proliferation, differentiation, stress reaction and apoptosis, mainly via p38 MAPK pathway, can induce apoptosis by adjusting the expression of several relevant proteins including p53 and Bcl-2 (Chang and Karin, 2001; Taylor et al., 2013; Hui et al., 2014; Zhang C. et al., 2014). Activity of the p38–MAPK pathway is significantly reduced in liver cancer cells and activation of p38 may induce apoptosis (Lamy et al., 2013). Bao HD et al. tested the anti-tumor effect of TP-1 in HepG2 and Huh7 HCC cells. Expression of Bax, Bcl-2 and survivin in the cells treated with TP-1 was obviously enhanced, leading to apoptosis of liver cancer cells due to activation of p38–MAPK pathway (Bao et al., 2016). A study by Xie et al. found that Huaier could mediate apoptosis of gastric cancer cells through the PI3K–AKT signaling pathway and prevent their proliferation by suppressing cyclin B1 expression and promoting G_2_/M-phase arrest (Xie H. X. et al., 2015).

Huaier granules can also increase the sensitivity of human hepatoma cell lines SKHEP-1 and HepG2 to rapamycin and cisplatin and thus enhance the anti-tumor effect of chemotherapeutics, probably by activating thr mTOR pathway (Hu et al., 2016). Huaier granules combined with DC-CIK showed increased efficacy in treating mice with HT-29 colon carcinoma cell line than either single method (Sun et al., 2017). Even so, the mechanisms of Huaier granules require further study.

### Shenqi fuzheng injection

Shenqi fuzheng injection (SFI) was approved by Chinese Food and Drug Administration in 1999 for clinical application (CFDA approval number, Z19990065). It mainly includes *Codonopsis pilosula* and *A. membranaceus* (ratio of 1:1) (Wang J. et al., 2014). Studies showed that SFI significantly suppressed tumor growth, neutralized toxicity of chemotherapeutics and increased immunity (Qi et al., 2015). Yang et al. conducted an animal experiment to treat nude mice induced by cyclophosphamide with SFI of low, medial and high doses (Wang J. et al., 2012). The results showed that SFI could dose-dependently increase the spleen index of mice, promote recovery of peripheral white blood cells and marrow cells, stimulate the proliferation of T cells and B cells, increase activity of splenic natural killer cells and peritoneal macrophages, and renew the level of serous IL-2. Many studies have indicated that SFI combined with systemic chemotherapeutics can generate a synergetic anti-tumor effect that improves the objective response rate (ORR), increases Karnofsky performance score (KPS) and immunity, and decreases the adverse event rate (Li J. et al., 2015; Zhang D. et al., 2017). In a meta-analysis of 15 random clinical trials (RCTs) among patients with late-stage gastric cancer, Yao et al. showed the ORR for patients treated with SFI and chemotherapy was significantly higher than for patients treated with chemotherapy alone (OR = 1.66, 95% confidence interval [CI]: 1.20–2.29, *P* < 0.05; Yao et al., 2014). Furthermore, KPS was significantly increased by the combinatorial treatment (OR = 3.74, 95% CI: 2.66–5.27, *P* < 0.05). Another meta-analysis that included a combined cohort of 722 patients with colon cancer found that the group treated with SFI combined with chemotherapeutics had a higher ORR and lower gastrointestinal toxicity than did the group treated with chemotherapy only. This result suggests that SFI can enhance the efficacy of chemotherapeutics while decreasing their side effects (Xu R. et al., 2017).

### Aidi injection

Aidi injection (CFDA approval number, Z52020236) is an anti-tumor Chinese medicine from the spotted jellyfish, *A. membranaceus*, Acanthopanax, and ginseng (Xiao et al., 2017). Aidi injections can reportedly alleviate side effects of chemotherapeutics and exert an adjuvant effect on the comprehensive treatment of DSM (Wang T. et al., 2014; Ge et al., 2016). In a meta-analysis of 32 RCTs by Wang et al., Aidi injections combined with chemotherapeutics improved the effective rate of chemotherapeutics for gastric cancer and the patient's quality of life, and decreased incidences of side effects such as nausea and vomiting, diarrhea, leukopenia III–IV, and thrombocytopenia III–IV (Jiancheng et al., 2015). Wang et al. treated patients with advanced colon cancer using Aidi injections combined with FOLFOX4 and found that the experimental group (*n* = 63) had a much lower rate of grade-II nausea, vomiting, and diarrhea than did the control group (*n* = 58) for 7 days (Wang J. et al., 2014).

### Kanglaite injection

Kanglaite injection is an anti-tumor medication mainly extracted from Chinese herb-coix seed (*Semen coicis yokuinin*), and has been approved by CFDA to treat gastric cancer, liver cancer, etc. (Qi et al., 2015). Kanglaite injection can reduce side effects of chemotherapy and radiotherapy, improve patients' quality of life, and reverse drug resistance to some extent. In a meta-analysis of Kanglaite injection combined with hepatic arterial intervention for the treatment of non-resectable HCC, the ORR and KPS of patients who received the combined treatments were both higher than for patients treated with a single method (Fu et al., 2014).

## Conclusions

In summary, we have reviewed the current anti-tumor mechanisms of TCM, including single herbal medicines (Table 1), Chinese herbal formulations, Chinese medicine preparations (Table 2), and TCM extract (Table 3), and their application in the comprehensive treatment of digestive system tumors (Table 4). TCM has a long history, and its application in many diseases, especially malignant tumors, has been widely reported (Wong et al., 2015; Xu et al., 2015; Chen X. et al., 2016; Zhang et al., 2016). However, due to the variety of medicinal plants (estimated over 12,000 species) and their complex components, TCM has not been widely recognized in Western countries (Chen et al., 2014). In recent years, with more extensive TCM investigations, continuous progress has been made in extraction technology, and standardization of TCM. The value of TCM in modern medical and health services is becoming more widely recognized. However, there are still some difficulties in the study of TCM for DSM, such as the determination of active substances and the accuracy of chemical composition determination (Zhang et al., 2018).

**Table 1:** Recent advances in anti-tumor mechanisms of single herbal medicines for digestive system malignancies.

**Cancer type**	**Single herbs (main active ingredients)**	**Object**	**Anticancer effects/mechanisms**	**References**
Liver cancer	*Astragalus membranaceus* (Astragalus Polysaccharide)	tumor xenograft model	Inhibits the growth of tumor by increasing Bax protein expression and decreasing Bcl-2 protein expression	Lai et al., 2017
Liver cancer	*A.membranaceus* (Astragalus Polysaccharide)	H22 cells	Induces the apoptosis cells by inhibiting the expression of Notch1	Huang et al., 2016
Liver cancer	*Sophora flavescens* (Oxymatrine)	HepG2 and SMMC-7721 cells	Inhibit cell proliferation and induce apoptosis by increasing expression of Bax and caspase 3, and decreasing expression of Bcl-2	Liu et al., 2016a
Liver cancer	*S.flavescens* (Matrine)	HepG2 and SMMC-7721 cells	Inhibits proliferation and induces apoptosis regulated by p53 inactivation through AMP-activated protein kinase (AMPK) signaling transduction	Xie S. et al., 2015
Liver cancer	*S.flavescens* (Matrine)	human cholangiocarcinoma cell lines (KMCH-1 and MzChA-1 cells)	Suppresses proliferation and induces apoptosis through suppression of JAK2/STAT3 signaling pathway.	Yang et al., 2015
Liver cancer	*S.flavescens* (Matrine)	Hep3B cells	Induces apoptosis by suppressing gene expression of minute double-mutant (MDM)2	Zhou et al., 2017
Pancreatic cancer	*S.flavescence* (Matrine)	HPAC and Capan-1 cells	Suppresses cell migration and invasion through down-regulating the expression of MT1-MMP via Wnt signaling	Ma et al., 2015
Gallbladder carcinoma	*S.flavescence* (Oxymatrine)	GBC-SD and SGC-996 cells, and tumor xenograft model	Inhibits cell proliferation and induces apoptosis through activation of caspase-3 and Bax, downregulation of Bcl-2 and nuclear factor κB	Wu et al., 2014
Gastric cancer	*A.membranaceus* (Astragalus saponins)	Human gastric adenocarcinoma cells	Induces apoptosis by activating caspase 3 and suppresses the process of angiogenesis by inhibiting the protein expression of VEGF, MMP-2 and MMP-9	Auyeung et al., 2012
Gastric cancer	*S.flavescence* (Matrine)	MKN45 cells	Inhibits cell growth through modulation of the NF-jB, XIAP, CIAP, and p-ERK proteins expression	Luo et al., 2012
Gastric cancer	*S.flavescence* (Matrine)	BGC823 cells	Inhibits cell migration and adhesion by affecting the structure and function of the vasodilator-stimulated phosphoprotein (VASP)	Zhang J. W. et al., 2013
Gastric cancer	*S.flavescence* (Oxymatrine)	MKN-45, BGC823, SGC7901 and HEK293 cells	Suppresses cell proliferation and invasion through inhibiting phosphorylation of EGFR (Tyr845)	Guo et al., 2015
Colorectal cancer	*A.membranaceus* (Astragalus saponins)	HT-29 cells	Induces the extrinsic apoptotic cascade and causes cell cycle arrest by modulation of both mTOR and ERK signaling pathways	Auyeung et al., 2010
Colorectal cancer	*S.flavescence* (Matrine)	HT29 cell	Inhibits cell apoptosis through the upregulation of Bax, the downregulation of Bcl-2, the release of Cyto C from the mitochondria to the cytosol and the activation of caspase-3 and caspase-9	Chang et al., 2013
Colorectal cancer	*S.flavescence* (Matrine)	LoVo cells	Inhibits proliferation and induces apoptosis by inactivating Akt pathway	Zhang S. et al., 2014
Colorectal cancer	*S.flavescence* (Oxymatrine)	RKO, HCT116, and SW480 cells	Suppresses cell invasion through inhibiting EMT via modulating NF-κB signaling pathway	Liang and Huang, 2016
Colorectal cancer	*S.flavescence* (Oxymatrine)	RKO cells	Inhibits cell migration via inhibition of PAI-1 and the TGF-β1/Smad signaling pathway	Wang M. et al., 2017

**Table 2:** Recent advances in anti-tumor mechanisms of Chinese medicine preparations for digestive system malignancies.

**Cancer type**	**Single herbs (main active ingredients)**	**Object**	**Anticancer effects/mechanisms**	**References**
Liver cancer	*Trametes robiniophila* Murr. (Huaier) (Huaier polysaccharide)	MHCC97-H cells	Inhibits metastasis through inactivating of the astrocyte elevated gene-1 (AEG-1)/epithelial–mesenchymal transition (EMT) pathway	Zheng et al., 2014
Liver cancer	*T.robiniophila* Murr. (Huaier) (Huaier polysaccharide)	HepG2 and Bel7402 cells	Induces cell apoptosis and S phase arrest via JNK signaling pathway	Zhang et al., 2015
Gastric cancer	*T.robiniophila* Murr. (Huaier) (Huaier polysaccharide)	MKN45 and SGC7901 cells	Inhibits cell proliferation by inhibiting cyclin B1 expression and induces cell apoptosis by modulating the PI3K/AKT signaling pathway in dose-dependent manner	Xie H. X. et al., 2015
Gastric cancer	*T.robiniophila* Murr. (Huaier) (Huaier aqueous extract)	SGC7901 and MGC803 cells	Suppresses cell metastasis and epithelial–mesenchymal transition (EMT) by Targeting Twist	Xu Z. et al., 2017
Colorectal cancer	*T.robiniophila* Murr. (Huaier) (Huaier aqueous extract)	T1 and T2 cells	Inhibits cell growth partially via downregulation of the Wnt/β-catenin pathway	Zhang T. et al., 2013

**Table 3:** Recent advances in anti-tumor mechanisms of traditional Chinese medicine extract for digestive system malignancies.

**Cancer type**	**Single herbs (main active ingredients)**	**Object**	**Anticancer effects/mechanisms**	**References**
Liver cancer	*Mylabris*(Cantharidin)	HepG2 hepatocellular carcinoma stem cells (HCSCs)	Inhibits cell proliferation through increasing expression of cdc2 (Tyr15) phosphorylation and induces apoptosis through regulating Wnt/β-catenin signaling pathway	Le et al., 2016
Liver cancer	*Mylabris*(Norcantharidin)	HepG2 cells and tumor xenograft model	Inhibits tumor growth by miR-214 modulating macrophage polarization	Lu et al., 2014
Liver cancer	*Mylabris*(Norcantharidin)	MHCC-97H, HepG2, Bel-7404, SMMC-7721, MHCC-97L, and SKHep-1 cells	Suppresses cell migration and invasion by up-regulating FAM46C expression via suppressing TGF-β/Smad signaling pathway	Wan et al., 2017
Liver cancer	*Coptis chinensis* (Berberine)	HepG2 cells	Induces apoptosis and autophagy death through inhibiting mTORC1 via AMPK activation	Yu et al., 2014
Liver cancer	*C.chinensis*(Berberine)	HepG2 cell line	Suppresses cell growth by regulating miR-22-3p targeting SP1	Chen J. et al., 2016
Liver cancer	*C.chinensis*(Berberine)	human CCA cell lines, KKU-213, and KKU-214 cells	Induces Cell Cycle Arrest through inhibiting of NF-κB and STAT3 Pathways via suppression of extracellular signal-regulated kinase (ERK) 1/2 action	Puthdee et al., 2017
Liver cancer	*C.chinensis* (Berberine)	SMMC-7721 and Bel-7402 cells	Suppresses cell invasion and migration by up-regulation of PAI-1 and down-regulation of uPA	Wang X. et al., 2016
Pancreatic cancer	*Mylabris* (Cantharidin)	PANC-1 and CFPAC-1 cells	Suppresses cell invasion by downregulation of MMP2	Shen et al., 2015
Pancreatic cancer	*Mylabris*(Cantharidin and Norcantharidin)	PANC-1, CFPAC-1 and pancreatic cancer stem cells	Impairs stemness of cells by repressing the β-catenin pathway and strengthen the cytotoxicity of the present therapeutics	Wang T. et al., 2015
Pancreatic cancer	*C.chinensis* (Berberine)	PANC-1 and MiaPaCa-2 cells	Inhibits cell growth through inhibiting mitogenic signaling via dose-dependent AMPK-dependent and independent pathways	Ming et al., 2014
Pancreatic cancer	*C.chinensis* (Berberine)	PANC-1 and MiaPaCa-2 cells	Induces apoptosis via ROS generation	Park et al., 2015
Gallbladder carcinoma	*Mylabris* (Norcantharidin)	GBC-SD cells and tumor xenograft model	Inhibits proliferation, invasion and migration by suppression of the PI3-K/MMPs/Ln-5γ2 signaling pathway	Zhang J. T. et al., 2014
Gastric cancer	*C.chinensis* (Berberine)	BGC-823 and SGC7901 cells, tumor xenograft model	Induces cell apoptosis by inhibiting the Akt/ mTOR/p70S6/S6 pathway	Yi et al., 2015
Gastric cancer	*C.chinensis* (Berberine)	MGC 803 cells and tumor xenograft model	Suppresses cell proliferation and tumorigenesis via inactivation of p38 MAPK	Li H. L. et al., 2016
Gastric cancer	*Mylabris* (Norcantharidin)	AGS cells	Induces apoptosis through caspase- and mitochondria-dependent signaling pathways	Zheng et al., 2016
Gastric cancer	*Mylabris*(Cantharidin)	SGC-7901 and BGC-823 cells	Induces G2/M phase arrest by regulating cycle-associated proteins and induces apoptosis by activating a caspase cascade or regulating the Bcl-2 family proteins.	Zhang C. et al., 2014
Colorectal cancer	*Mylabris*(Norcantharidin)	HT29 and HCT116 cells	Inhibits cell growth by suppressing the expression and phosphorylation of both EGFR and c-Met	Qiu et al., 2017
Colorectal cancer	*Mylabris*(Norcantharidin)	HT-29 cells and tumor xenograft model	Inhibits tumor growth and lymphangiogenesis by directly or indirectly downregulating VEGF-A,-C,-D/VEGFR-2,-3 signaling pathways	Li X. P. et al., 2015
Colorectal cancer	*C.chinensis* (Berberine)	SW620 and LoVo cells, tumor xenograft model	Inhibits invasion and metastasis of cells via down-regulation of COX-2/PGE2- JAK2/STAT3 signaling pathway.	Liu et al., 2015

**Table 4:** Herbal medicine combined with chemotherapeutics for digestive system malignancies.

**Cancer type**	**Herbal medicine**	**Chemotherapeutics**	**Object**	**Main findings**	**References**
Liver cancer	*A.membranaceu* (Astragalus polysaccharides)	Adriamycin	tumor xenograft model	Astragalus polysaccharides exerted a synergistic anti-tumor effect with Adriamycin through enhancing the expression of IL-1α, IL-2, IL-6, and TNF-α, decreasing IL-10, and down-regulating MDR1 mRNA and P-GP expression levels	Tian et al., 2012b
Liver cancer	*A.membranaceu* (Astragalus polysaccharides)	Cyclophosphamid, Adriamycin, 5-Fluorouracil, Cisplatin, etoposide, and Vincristine	H22/ADM cells	Astragalus polysaccharides exerted a synergistic anti-tumor effect with chemotherapeutics through decreased the expression of the MDR1 protein mediated by downregulation of MDR1 mRNA expression and/or inhibition of P-GP efflux pump function	Tian et al., 2012a
Liver cancer	*A.membranaceu* (Astragaloside II)	5-Fluorouracil	Bel-7402 and Bel-7402/FU cells	Astragaloside II sensitized cells to 5-fluorouracil-induced cell death via inhibition of pro-survival autophagy involvement of MAPK-mTOR pathway	Wang G. et al., 2018
Liver cancer	*S.flavescens* (Oxymatrine)	5-Fluorouracil	HepG2 and SMMC-7721 cells, tumor xenograft model	Oxymatrine sensitized HCC to 5-Fu treatment by suppression of ERK activation through the overproduction of ROS	Liu et al., 2016b
Liver cancer	*T.robiniophila* Murr. (Huaier) (Huaier aqueous extract)	Rapamycin or Cisplatin	SKHEP-1 and HepG2 cells	Huaier aqueous extract inhibited tumorigenic capacity of cells increased sensitivity of cells to chemotherapeutics with activated mTOR *in vivo*	Hu et al., 2016
Pancreatic cancer	*Mylabris* (Cantharidin and Norcantharidin)	Tamoxifen	PANC-1, BxPC-3, CFPAC-1, Capan-1, PL-45 and SW-1990 cells	Tamoxifen increased the cytotoxicity mediated by cantharidin and norcantharidin through inhibiting the protein kinase C (PKC) phosphorylation	Xie X. et al., 2015
Pancreatic cancer	*C.chinensis* (Berberine)	Gemcitabine	BxPC-3, Capan-2, MIA PaCa-2, and PANC-1 cells	Berberine potentiated gemcitabine sensitivity by down-regulating STAT3/NF-kB signaling	Gong et al., 2017
Gastric cancer	*C.chinensis* (Berberine)	Cisplatin	SGC-7901, BGC-823, SGC-7901/DDP and BGC-823/DDP cells	Berberine reduced cisplatin resistance of gastric cancer cells by modulating the miR-203/Bcl-w apoptotic axis	You et al., 2016
Gastric cancer	*C.chinensis* (Berberine)	5-Fluorouracil	Human gastric adenocarcinoma cells	Berberine enhanced 5-Fluorouracil sensitivity by a synergistic inhibition of survivin and STAT3 level.	Pandey et al., 2015
Gastric cancer	*C.chinensis* (Berberine)	erlotinib and cetuximab	MKN45, BGC823 and SGC7901 cells	Berberine enhanced the anti-tumor activity of erlotinib and cetuximab by inhibiting EGFR signaling pathway	Wang J. et al., 2016
Colorectal Cancer	*S.flavescens* (Oxymatrine)	Oxaliplatin	HT29 and SW480 cells, tumor xenograft model	Oxymatrine enhanced antitumor activity of oxaliplatin through PI3K/AKT/mTOR pathway	Liu et al., 2016c
Colorectal Cancer	*S.flavescens* (Oxymatrine)	Irinotecan	HT29 cells	Oxymatrine exerted a synergistic anti-tumor effect with Irinotecan by up-regulation of the TOPO I, Bax and Caspase-3 protein expression	Duan et al., 2017
Colorectal Cancer	*A.membranaceu* (Astragaloside IV)	Cisplatin	HCT116 and SW480 cells	Astragaloside IV increased Cisplatin chemosensitivity of colorectal cancer cells partly via suppressing the expression of NOTCH3	Xie et al., 2016
Colorectal Cancer	*A.membranaceu* (Astragaloside IV)	Oxaliplatin	SW-480 cells	Astragaloside IV increased the sensitivity to chemotherapy through inhibiting EMT induced by miR-134 expression.	Ye et al., 2017
Colorectal Cancer	*A.membranaceu* (Astragalus saponins)	Vinblastine	HCT 116, DLD-1 and LoVo cells, tumor xenograft model	Astragalus saponins exerted a synergistic anti-tumor effect with Vinblastine by downregulating expression of key proangiogenic and metastatic factors including VEGF, bFGF, metalloproteinase (MMP)-2, and MMP-9	Auyeung et al., 2014

Unlike Western medicine, the anti-metastatic effects of TCM reflect comprehensive treatment of multiple targets and components, which can inhibit the various links of metastasis from many aspects, with less toxicity. Although some progress has been made in basic research and clinical application of TCM in the treatment of DSM, many components and mechanisms still need to be clarified. Modern scientific and technological means are being used to study anti-tumor mechanisms of TCM at the cellular, molecular and genome levels, to make full use of TCM in the management of digestive system malignancies.

## Author contributions

JS, ZC, XZ and YX wrote the manuscript. JS, WY, YL and RC provided the critical revisions. All authors approved the final version of the manuscript for submission.

### Conflict of interest statement

The authors declare that the research was conducted in the absence of any commercial or financial relationships that could be construed as a potential conflict of interest.

## Supplementary material

Supplementary Figure 1 Chemical structure of cantharidin and norcantharidin.

Supplementary Figure 2 Chemical structure of berberine.
