# Supplementary figures and images for: Recent Advances in Herbal Medicines for Digestive System Malignancies
Source: Front Pharmacol. 2018 Nov 20;9:1249. doi: 10.3389/fphar.2018.01249 (PMC6256117; doi:10.3389/fphar.2018.01249)

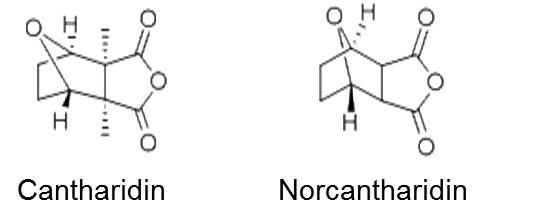

Supplement: Supplementary Figure 1 — Chemical structure of cantharidin and norcantharidin. [file Image_1.JPEG]

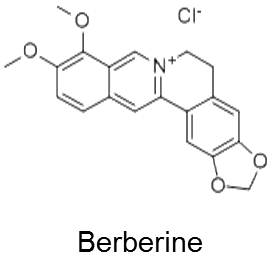

Supplement: Supplementary Figure 2 — Chemical structure of berberine. [file Image_2.JPEG]
